# METRIC (MREnterography or ulTRasound in Crohn’s disease): a study protocol for a multicentre, non-randomised, single-arm, prospective comparison study of magnetic resonance enterography and small bowel ultrasound compared to a reference standard in those aged 16 and over

**DOI:** 10.1186/1471-230X-14-142

**Published:** 2014-08-11

**Authors:** Stuart Taylor, Susan Mallett, Gauraang Bhatnagar, Stuart Bloom, Arun Gupta, Steve Halligan, John Hamlin, Ailsa Hart, Antony Higginson, Ilan Jacobs, Sara McCartney, Steve Morris, Nicola Muirhead, Charles Murray, Shonit Punwani, Manuel Rodriguez-Justo, Andrew Slater, Simon Travis, Damian Tolan, Alastair Windsor, Peter Wylie, Ian Zealley

**Affiliations:** 1 Center for Medical Imaging, University College London, 250 Euston Rd, London NW1 2PG, UK; 2 Medical Statistics, Department of Primary Health Care Sciences, University of Oxford, 2nd Floor Offices, 23-38 Hythe Bridge Street, Oxford, UK; 3 Department of Gastroenterology, University College London Hospital, 235 Euston Road, London, UK; 4 Intestinal Imaging, St Marks Hospital,Harrow Road, London, UK; 5 Gastroenterology,Leeds Teaching Hospitals NHS Trust, St James’s University Hospital, Beckett Street, Leeds, UK; 6 Gastroenterology, St Marks Hospital, Harrow Road, London, UK; 7 Medical Imaging, Queen Alexandra Hospital, Southwick Hill Road, Cosham, UK; 8 Public representative, Patient forum, National Association of Crohn’s and colitis, c/oUCL Partners CTU, Maple House, 149 Tottenham Court Rd, London, UK; 9 Health Economics, UCL Research Department of Epidemiology and Public Health, University College London, 1-19 Torrington Place, London, UK; 10 UCL Clinical Trials Unit, UCL Gower Street, London, UK; 11 Gastroenterology, Royal Free Hospital, Pond Street, London, UK; 12 Department of Gastrointestinal Pathology, University College London Hospital, 235 Euston Road, London, UK; 13 Medical Imaging, Oxford University Hospitals NHS Trust, Oxford, OX3 9DU, UK; 14 Translational Gastroenterology Unit, Oxford University Hospitals NHS Trust, Oxford, OX3 9DU, UK; 15 Clinical Radiology, Leeds Teaching Hospitals NHS Trust, St James’s University Hospital, Beckett Street, Leeds, UK; 16 Department of Surgery, University College London Hospital, 235 Euston Road, London, UK; 17 Imaging, Royal Free Hospital, Pond Street, London, UK; 18 Medical Imaging, Ninewells Hospital, Dundee, UK

**Keywords:** Crohn’s disease, Inflammatory bowel disease, MRE, USS, Consensus panel

## Abstract

**Background:**

Crohn’s disease (CD) is a lifelong, relapsing and remitting inflammatory condition of the intestine. Medical imaging is crucial for diagnosis, phenotyping, activity assessment and detecting complications. Diverse small bowel imaging tests are available but a standard algorithm for deployment is lacking. Many hospitals employ tests that impart ionising radiation, of particular concern to this young patient population. Magnetic resonance enterography (MRE) and small bowel ultrasound (USS) are attractive options, as they do not use ionising radiation. However, their comparative diagnostic accuracy has not been compared in large head to head trials. METRIC aims to compare the diagnostic efficacy, therapeutic impact and cost effectiveness of MRE and USS in newly diagnosed and relapsing CD.

**Methods:**

METRIC (ISRCTN03982913) is a multicentre, non-randomised, single-arm, prospective comparison study. Two patient cohorts will be recruited; those newly diagnosed with CD, and those with suspected relapse. Both will undergo MRE and USS in addition to other imaging tests performed as part of clinical care. Strict blinding protocols will be enforced for those interpreting MRE and USS. The Harvey Bradshaw index, C-reactive protein and faecal calprotectin will be collected at recruitment and 3 months, and patient experience will be assessed via questionnaires. A multidisciplinary consensus panel will assess all available clinical and imaging data up to 6 months after recruitment of each patient and will define the standard of reference for the presence, localisation and activity of disease against which the diagnostic accuracy of MRE and USS will be judged. Diagnostic impact of MRE and USS will be evaluated and cost effectiveness will be assessed. The primary outcome measure is the difference in per patient sensitivity between MRE and USS for the correct identification and localisation of small bowel CD.

**Discussion:**

The trial is open at 5 centres with 46 patients recruited. We highlight the importance of stringent blinding protocols in order to delineate the true diagnostic accuracy of both imaging tests and discuss the difficulties of diagnostic accuracy studies in the absence of a single standard of reference, describing our approach utilising a consensus panel whilst minimising incorporation bias.

**Trial registration:**

METRIC - ISRCTN03982913 – 05.11.13.

## Background

Crohn’s disease (CD) is an inflammatory condition, with a wide spectrum of intestinal manifestations ranging from superficial bowel wall ulceration to deep penetrating disease, characterised by fistulae and abscesses. Over time, repeated inflammatory insults can result in the development of fibrosis and stricture formation. It affects 200,000 people in the UK (around 1 in 500), most are young (diagnosed < 35 years) and the costs of direct medical care in the UK exceed £500 million [1]. A range of potentially toxic medical treatment options, such as immune-modulators, or targeted surgical interventions are currently employed in disease management. The optimal treatment strategy requires accurate assessment of disease presence, extent, activity and complications. CD typically affects the small bowel, most of which is beyond the reach of conventional colonoscopy. Small bowel imaging therefore plays a vital role in diagnosing and phenotyping CD, thereafter assessing disease activity and complications.

At present there is no single imaging modality that has been proven universally superior in either suspected or established CD. A plethora of small bowel investigations are currently performed within the NHS, approximately 100,000 each year, including Barium fluoroscopy (BaF), Computerised Tomography (CT), Ultrasound (USS) and Magnetic Resonance Enterography (MRE). According to a UK survey in 2010 [2], 90% of NHS radiology departments routinely perform BaF to investigate patients with known or suspected CD, 80% perform CT, 56% USS and 38% MRI. Small bowel imaging tests differ in their individual attributes; for example BaF affords high quality assessment of the bowel mucosa, whilst cross sectional techniques such as CT, MRE and USS facilitate evaluation of the bowel wall and extra-enteric tissues. An important attribute is the use or otherwise of ionising radiation. The currently most used tests, BaF and CT, impart a significant radiation dose. This is concerning given that CD patients are young and usually undergo serial imaging to assess disease evolution over their lifetime. An audit in 2007 found 15.5% of CD patients received a cumulative radiation dose that may increase cancer risk by 7.3% [3]. USS and MRE are alternatives that do not use ionising radiation but deployment in the NHS is currently ad hoc. The choice of small bowel imaging investigation currently depends largely on non-evidence based decision-making, such as clinician personal preference, perceived costs, available infrastructure and radiological expertise.

Three systematic reviews have been published to date evaluating the accuracy of imaging tests in the diagnosis of CD and for assessing disease activity [4-6]. All have highlighted marked heterogeneity in the available literature, with most studies being single centre and involving relatively small patient numbers. Variation in the applied standard of reference between studies is also apparent. The largest systematic review [6] incorporated 68 studies, and compared the performance of CT, MRI and USS for diagnosis, and disease activity classification. For diagnosis of disease location sensitivity of USS ranged from 75 to 93% and for MRI from 77 to 91%. Specificity ranged from 98 to 100% (USS) and 60 to 100% (MRI). The diagnostic accuracy to detect active disease per patient of studies USS sensitivity and specificity for detecting active disease was 85% (range 75 to 100%) and 91% (range 82-100%) respectively and for MRI were 80% (range 78-100%) and 82% (range 46%-100%) respectively. Only one study used the recommended direct diagnostic test comparison study design shown to reduce bias by assessing the same patients with multiple tests [7]. Miao et al. [8] reported of sensitivity 87% for both USS and MRI and specificities of 100% (7/7 patients) and 71% (5/7 patients) respectively in a study with 30 patients (23 with Crohn’s and 7 with no disease) [8].

Ultimately, the optimal imaging strategy for CD remains uncertain and single centre data is of limited utility. Unbiased, robust data to inform the implementation strategy for newer imaging technologies are currently unavailable, although international guidelines on imaging advocate MRE for diagnosis and USS for assessing disease activity, where resource and expertise are available [9].

In this article, we describe the protocol for the METRIC study (ISRCTN03982913), a multicentre, non-randomised, single-arm, prospective comparison study of MRE and USS in newly diagnosed CD, or established disease with suspected relapse. The sample size of our study is ten times larger than the only other study directly comparing MRE and USS in the same patients (8). Participating radiologists are members of BSGAR, British Society of Gastrointestinal and Abdominal Radiology. Research on this topic has been commissioned and funded by the UK Health Technology Assessment (HTA) programme (11/23/01). The full protocol adheres to the principals of the SPIRIT guidelines for clinical trials protocols [10].

### Study objectives

•Directly to compare the diagnostic accuracies of MRE and USS for detecting small bowel CD, and grading of inflammatory activity. This will include subgroup analysis of patients with a new diagnosis of CD and those suffering a relapse. The reference standard consists of a consensus panel, with collective review of all the available clinical and imaging data over a 6-month follow up period.

•Directly to compare diagnostic accuracies of MRE and USS for detecting colonic CD in those undergoing colonoscopy.

•To use a novel trial design to reduce uncertainty in evaluating the impact of MRE, USS and conventional imaging methods by direct capture of patient management.

•To evaluate the cost effectiveness of MRE and USS compared to each other, and to conventional imaging methods.

## Methods

### General

**Figure 1:**
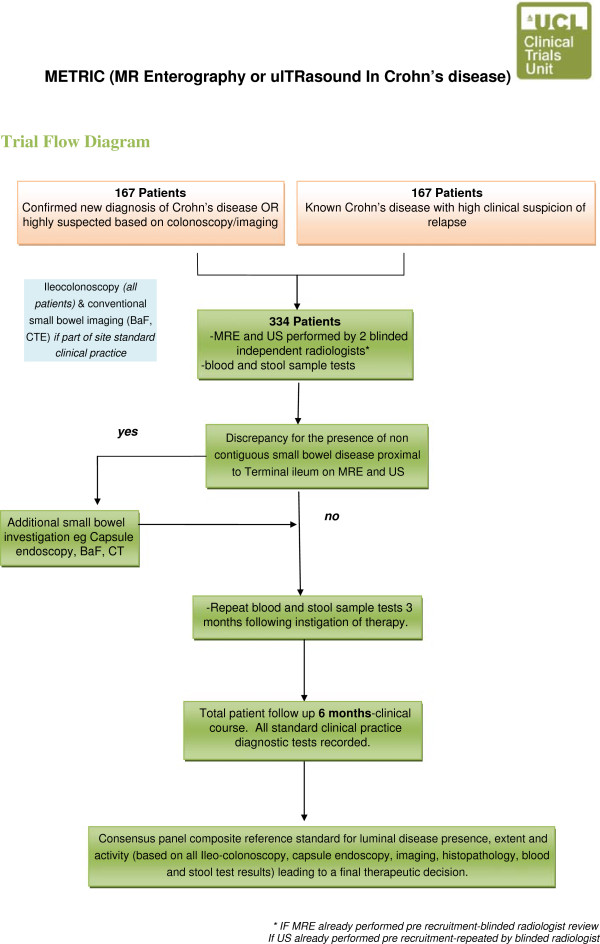
Flow diagram outlining the stages of the METRIC trial.

This is a multi-centre prospective cohort study comparing the diagnostic accuracy of MRE and USS for the presence, extent and activity of small bowel Crohn’s disease. The trial framework is to detect superiority of MRE over USS (Figure 1).

### Inclusions/exclusion criteria

The trial will recruit from two defined patient cohorts: (1) newly diagnosed CD patients (or within 3 months of diagnosis) and (2) those with previously confirmed CD with a high clinical suspicion of luminal relapse, requiring radiological investigation.

In the new diagnosis cohort, eligible patients are aged 16 years or older, have given written informed consent, undergoing or having undergone colonoscopy, and either:

•Newly diagnosed (within 3 months) with CD based on endoscopic, histological, clinical and radiological findings, [11] or

•Highly suspected of CD based on characteristic endoscopic, imaging and/or histological features but pending final diagnosis.

In the suspected relapse cohort, eligible patients are aged 16 years or older, able to give written informed consent, with a known diagnosis of CD together with a high clinical suspicion of luminal relapse defined as:

•Objective markers of inflammatory activity (raised CRP >8 mg/L OR raised calprotectin > 100 mcg/g), or

•Symptoms suggestive of luminal stenosis (including obstructive symptoms such as colicky abdominal pain, vomiting) or abnormal endoscopy suggesting relapse.

Patients with any psychiatric or other disorder likely to affect on informed consent and those with evidence of severe or uncontrolled systemic disease, which at the principal investigator’s discretion renders the patient unsuitable for participation in the study, will be excluded. Pregnant patients or those with contraindications to MRE (e.g. allergy to all suitable contrast agents, cardiac pacemaker, severe claustrophobia, or an inability to lie flat) are also excluded. Recruited patients with suspected disease whose final diagnosis is not CD, and those who undergo surgical resection prior to colonoscopy will also be excluded.

### Ethical arrangements and consent

The METRIC trial achieved National Health Service research ethics committee (NHS REC) approval in September 2013 (13/SC/0394) and is being conducted in accordance with the principles of Good Clinical Practice (GCP). Informed consent is a prerequisite. University College London’s Clinical Trials Unit supervises the trial.

### Diagnostic interventions

#### MRE

Recruited patients will undergo MRE at their recruitment site. The usual site radiographer team will perform the examination providing they are deemed competent by the site radiology lead.

The MRI platform (i.e. manufacturer and Tesla (T) strength) will be decided by the local radiologist according to scanner availability and usual practice. It is anticipated most MRE will be performed at 1.5 T. Exact imaging parameters will vary according to MRI platform but a minimum dataset of sequences will be acquired (Table 1). The choice of oral contrast prior to MRE will also be according to the usual practice of the recruitment site.

**Table 1:** MRE protocol outlining the minimum and optional MRI sequences that may be performed

Minimum	Coronal true FISP
	Buscopan-20 mg IV
	Axial and coronal non Fat Sat HASTE
	Coronal Fat Sat HASTE
	Axial diffusion b values 50 and 600
	Coronal pre and post gadolinium T1 (60–70 sec)
Optional	Axial True FISP
	Axial Fat Sat HASTE
	Axial post gadolinium T1
	True FIP dynamic Motility

In some patients MRE will have been performed as part of usual clinical care prior to recruitment. If it has been acquired within the preceding 4 weeks according to the minimum dataset of sequences, the MRE will be eligible for inclusion in the trial and will not need to be repeated.

#### USS

Recruited patients will also undergo small bowel USS at their recruitment site. This will be performed by a radiologist or trained sonographer fulfilling competency criteria (see section below). For the purposes of the trial, patients will not receive any oral agent before the USS other than an optional 2 cups of water to improve visualisation of the duodenum. The local radiologist will select the USS platform according to scanner availability and usual practice. Exact imaging parameters will vary according to USS platform but a minimum probe frequency and examination technique will be required (Table 2).

**Table 2:** USS imaging protocol

Preparation	Nil by mouth- 4 hours
Technical requirements	Use of both curve-linear and high resolution probe (min 5 Mhz frequency)
Procedure	Systematic review of colon and small bowel with both probes
	Review of enteric tissues
	Application of colour Doppler (typical flow 6-9 m/s)

USS acquired up to 4 weeks prior to recruitment will be repeated unless full blinding of the original performing radiologist or sonographer to clinical and imaging data can be assured.

### Other small bowel imaging

Many recruited patients will also undergo conventional small bowel imaging as part of usual clinical care, notably BaF, CT enterography and in some cases capsule endoscopy. The results of these tests will provide at least one independent small bowel imaging test for the consensus reference standard for the proximal small bowel, or non-endoscopically visualised terminal ileum.

In cases where the MRE and USS are discrepant for the presence of disease and no other small bowel imaging has been performed, a third arbiter test will be conducted. Discrepancy will be defined as disagreement between MRE and USS for the presence of disease in non-endoscopically visualised terminal ileum, or for the presence of disease in the proximal bowel upstream of the terminal ileum. The choice of arbiter small bowel imaging test will be at the discretion of the local recruitment site.

### Radiologist competence and training

A network of UK NHS hospitals with lead radiologists affiliated to the British Society of Gastrointestinal and Abdominal Radiology (BSGAR) will be used, ensuring appropriate imaging expertise for the purposes of the study.

All radiologists reporting on the trial are post-FRCR with at least one year of sub-specialty GI experience. They have previous experience of MRE and USS that has been supplemented with a two-day training course for the purposes of the study. Participating sonographers will already be performing USS in clinical practice, having attended a trial specific workshop and be deemed competent by their supervising radiologist Trial centres encompass both teaching and district general hospitals to enhance generalisability.

### Blinding of trial imaging

Unbiased estimates of imaging test diagnostic accuracy can only be achieved if those interpreting the tests are unaware of the findings of contemporaneous imaging and endoscopy. For example a radiologist aware of endoscopically confirmed terminal ileal disease could not give an unbiased evaluation of subsequent USS or MRE in the same patient. Similarly, interpretation of MRE or USS could be influenced by knowledge of the results of the other test. Thus, detailed instructions for satisfactory blinding of radiologists have been outlined in the protocol. Notably, both MRE and USS will be interpreted by different radiologists blinded to all clinical information other than the patient cohort (relapse or new diagnosis) and surgical history. If blinding of the original reporting radiologist cannot be assured, MRE images will be re-analysed by a blinded local or central radiologist (as appropriate), and USS will be repeated.

### Reporting of trial imaging

A case report form (CRF) will be generated for MRE and USS in all recruited patients. The CRF will detail the technical quality of the examination, together with the presence, extent and activity of Crohn’s disease. For the purposes of data recording, the bowel will be divided into duodenum, jejunum, ileum, terminal ileum and colon (rectum, sigmoid, descending colon, transverse, ascending and caecum). Colonic segments will be defined using previously published definitions [12].

For each segment, radiologists will indicate the presence or absence of Crohn’s disease together with their diagnostic confidence on a 6 point scale. Data on the length of disease, activity, the presence of functionally significant stenosis, and extra-enteric complications such as abscess or fistulae will also be recorded. Standard definitions will be used for the identification of Crohn’s disease [13,14]. All distinct sections of disease in a segment will be recorded separately.

Disease activity on MRE and USS will be assessed using published validated criteria [13]. Reporting radiologists will state if, in their opinion and based on these criteria, any disease present is active or non-active on a (per) segment(al) and per patient basis. Reporting radiologists will also record the impact of certain additional MRE sequences, such as diffusion and contrast enhancement to their decision making.

### Experience questionnaires

Patients recruited prior to the MRE will be issued a questionnaire pertaining to the acceptability of the oral contrast preparation, immediately before the MRE, during the test, and up to 48 hours later. Patients will be questioned on their experience of other test facets such as scanner noise.

A second questionnaire will be administrated to all recruited patients and will assess comparative experience and acceptability of MRE, USS and any other imaging tests undergone during their clinical care.

### Evaluating therapeutic impact

An assessment of the impact of MRE and USS on diagnostic confidence and patient management compared to conventional imaging will be evaluated.

Gastroenterologists at each site will review the clinical data and record their diagnostic confidence for the presence and location of CD, its activity, extra-enteric complications, need for additional investigations and planned therapeutic strategy using a previously published proforma [2]. A radiologist will then present the findings of one of the imaging modalities (MRE, USS or more conventional imaging such as CT, BaF (if performed), and the gastroenterologist will re-complete the proforma in light of these imaging findings, noting changes (if any) in their diagnosis, diagnostic confidence or therapeutic decision. After 4 weeks, the process will be repeated, although the radiologist will present another imaging modality. The order of revelation of the imaging modalities for each individual patient will be randomised centrally. The process will be repeated until all 3 modalities have been revealed.

### Reference standard

There is no single reference standard that can uniformly be employed for the phenotyping of CD. Diagnosis and phenotyping in clinical practice is made on a combination of clinical, endoscopic, imaging, histopathological and biochemical criteria. The HTA has given guidance regarding the evaluation of diagnostic tests when there is no “gold standard” [15]. The current trial will use the construct reference standard paradigm (panel diagnosis) incorporating the concept of clinical test validation. Specifically patients’ clinical course will be followed for six months after recruitment, during which time the findings of the MRE and USS will have been acted upon by clinicians and incorporated into their therapeutic decision-making.

Ileo-colonoscopy (combined with histological assessment of tissue biopsies) is considered the most robust standard of reference for diagnosis and phenotyping of CD within the colon and terminal ileum (last few centimetres of small bowel). All newly diagnosed patients will have undergone ileo-colonoscopy as part of their normal clinical care.

Consenting patients will have their Harvey Bradshaw index, plasma CRP and faecal calprotectin measured at recruitment and after 3 months. These data will be made available to the panel to provide an objective measure of disease activity.

Each recruitment site will convene their own consensus panel, to derive the reference standard for disease presence, extent and activity at the time of the trial imaging in recruited patients. The panels will consider all available clinical information including the results of conventional investigations, endoscopy (conventional and capsule), MRE, USS, surgical findings, histopathology (surgical resection and biopsies), HBI, CRP, and FC (and changes thereof in response to therapy), follow up imaging and clinical course. Each panel will consist of at least one (and ideally two) gastroenterologists and two radiologists (one local to the site and one external). A histopathologist will be available to the panel if required. When defining the reference standard for the primary outcome, the panel will record their confidence in the findings of each contributing test (e.g. all available imaging tests, endoscopy etc.) to allow assessment of incorporation bias. Each panel will complete the final reference standard CRF against which the diagnostic accuracy of imaging tests will be compared.

### Cost effectiveness

Resource use data for the main drivers of hospital costs will be collected using a study-specific CRF. Additionally, resource use diaries will be administered to all patients at consent and then once more at 3 months. The diaries will be used to collect data on primary and community care contacts for the 6 month period of follow up from recruitment. Economic costs associated with ultrasound and MRE will be extrapolated.

### Outcome measures

#### Primary outcome measure

Difference in per patient sensitivity between MRE and USS for the correct identification and localisation of small bowel CD.

The sensitivity of each test to detect presence of disease (both active and inactive disease) in the correct location is measured against the reference standard, consensus panel review at 6 months. There will be subgroup analysis for separate population of new diagnosis versus relapse patients.

#### Secondary outcome measures

1. Difference in per patient specificity of MRE and USS for the correct identification and localisation of small bowel CD.

2. Comparison of USS and MRE detection of patients with active small bowel CD.

This will include:

a. Difference in sensitivity and specificity per patient based on a consensus

review as a reference standard

b. Difference in sensitivity and specificity in the terminal ileum in those patients undergoing terminal ileostomy as a reference standard

c. Additional analysis for colonic segments in patients with a colonoscopic reference examination

3. Comparison of USS and MRE diagnostic accuracy to detect presence of disease (either active or inactive)

a. Difference in sensitivity and specificity per patient in small bowel and colonic CD

b. Difference in sensitivity and specificity of terminal ileum segment in subgroup of patients undergoing colonoscopy in small bowel and colonic CD

c. Difference in sensitivity and specificity per segment in subgroup of patients undergoing colonoscopy in colonic CD

d. Subgroup analysis of (i) and (ii) in patients with small bowel only

4. Comparative impact of MRE and USS on clinician diagnostic confidence for the presence of CD and influence on patient management

5. The lifetime incremental cost and cost-effectiveness of assessment using MRE or USS

For the above there will be subgroup analysis for separate populations of new diagnosis versus relapse patients.

In addition several substudies are planned including

1. Diagnostic accuracy and radiologist confidence using hydrosonography compared to conventional USS

2. Comparative patient experience of MR and USS

3. Diagnostic impact of novel MRE sequences, notably diffusion weight imaging on disease detection, diagnostic confidence and disease activity assessment

4. Inter-observer variation in the evaluation of MRE and USS datasets by radiologists, to assess the impact of diagnostic confidence on accuracy

### Sample size

Power is based on the primary outcome stipulated by the HTA: diagnostic accuracy for CD extent. There are two aspects to correctly assigning disease extent; correctly detecting the presence of disease and correctly assigning its segmental location. For example a test which correctly identifies disease in the terminal ileum of the small bowel, but misses disease in the proximal bowel (e.g. jejunum) will conceivably result in an incorrect patient management decision i.e. such a test would be inaccurate for defining the extent of CD. Power is thus based on a two facetted compound accuracy measure (disease presence and disease location) [4,16,17].

A total cohort of 301 (210 patients with disease) is required to detect a 10% superiority of MRE over USS in correctly assigning disease presence and location at 90% power (type 2 error) [18]. Allowing 10% loss to follow up (referring to patients who fail to undergo the complete set of initial imaging – colonoscopy, ultrasound and MRI for new diagnosis patients and ultrasound and MRI for relapse patients – currently this lies at 12%), the total cohort is of 334 patients (167 new diagnosis patients and 167 relapse patients).

### Analysis

A detailed statistical analysis plan will be produced and finalised prior to data lock and transfer to trial statistician. Analysis will be based on all patients in the study. The primary and secondary outcomes will be based on available case analysis with a sensitivity analysis using multiple imputations, best case and worst case analysis.

Analysis for the primary outcome will use logistic regression of paired binary outcomes for comparison of diagnostic accuracy measures of MRE and USS within patients, allowing adjustment for clustering by centre. 95% confidence intervals will be calculated and p-values of <0.05 will be considered statistically significant. A similar approach will be used for the secondary outcomes.

There will be no adjustment of p-values for secondary outcomes for multiple testing. STATA statistical software will be used.

## Discussion

### Blinding of trial imaging

Ascertaining the true standalone diagnostic accuracy of an individual imaging test is only possible in the absence of external influences to radiological decision making. Interpretation of MRE or USS is likely to be influenced by knowledge of clinical parameters and findings of other imaging tests. The study methodology has therefore been specifically designed to ensure strict blinding of the radiologists. Radiologists’ information is restricted to the patient cohort (new diagnosis or relapse), and the history of previous surgery; this information would be available in usual clinical care and withholding it would not reflect routine clinical practice. Each recruitment site has identified two participating radiologists so the MRE and USS for each recruited patient can be conducted and interpreted by an independent radiologist. Trial MREs will be reviewed on workstations separate from the hospital reporting platforms, to ensure blinding from other imaging or reports. Similarly, during USS, where interpretation is more “real time”, the radiologist performing USS will be isolated from all material usually available in the clinical setting. The patient and the radiologist will be advised not to converse with the patient regarding current diagnosis during the USS and where feasible the patient will accompanied by the research nurse during the USS.

For those patients who underwent an MRE prior to recruitment, their images will be re-evaluated by an alternate radiologist, blinded to other patient information. For patients who underwent an USS prior to recruitment, their USS will be repeated by an alternate radiologist (unless appropriate blinding of the radiologist performing the original USS can be assured).

### Consensus panel as reference standard and assessment of incorporation bias

There is no single test proven to be a reference standard in the diagnosis of disease presence, extent and activity in CD within the small bowel. In such cases, a consensus panel may be convened to judge the presence or absence of the target condition based on multiple sources of information, as recommended by the HTA [15].

There is significant variation in the construct and behaviour of consensus panels. A recent systematic review of published methods and reporting of studies using expert panels to define the reference standard in diagnostic studies provides recommended options for consensus panels composition, decision making and reporting [19]. One such crucial point is whether or not to incorporate the index tests, MRE and USS, in the consensus review. Inclusion of the index test result may overestimate its importance, leading to incorporation bias, and falsely high accuracy. Conversely, excluding the index test may hamper the ability to make the correct diagnosis, resulting in misclassification of the disease status. Several staged approaches have been proposed, such as those in which the panel forms an opinion without the index tests and these are then revealed for review and a final decision is reached. Such approaches are time and personnel intensive and deemed impractical on such a large scale. In order to minimise incorporation bias, the expertise of all panel members will be recorded as well as the level of consensus reached (majority or unanimous) for the primary outcome. The panel may request review by a second independent panel if they are unable to reach consensus on the primary outcome reference standard (presence and location of small bowel CD).

When defining the reference standard for the primary outcome, the panel will record their confidence in the findings of each contributing test (e.g. all available imaging tests, endoscopy etc.). Specifically they will state if the normality or otherwise of each available test is clear cut or equivocal. Such data will help detect potential incorporation bias (e.g. if an equivocal MRI “overrides” a clear cut CT enterography and USS). The trial statistician and CI will review the outcome of the panel review for the first 50 recruited patients centrally. If incorporation bias is deemed problematic, a decision will be made as to the need for a routine second panel review of cases when the findings of tests contributing to the primary outcome reference are discrepant.

To standardise the decisions of the consensus panel a member of the central trial team will attend each consensus meeting to ensure similar criteria are used in defining disease extent.

The METRIC trial is a multi-centre prospective cohort study comparing the diagnostic accuracy of MRE and USS for the presence, extent and activity of small bowel Crohn’s disease. The lack of a single reference test has led to the use of a consensus panel to act as the reference standard, with specific attention to minimize incorporation bias. The trial methodology includes detailed protocols in order to ensure adequate blinding of radiologists. In addition the trial includes direct recording of patient management decisions resulting from MRE and USS test results, to reduce uncertainty in evaluating the comparative impact of modalities. The sample size of our study is ten times larger than the only other study directly comparing MRE and USS in similar patients (8).

### Trial status

The trial was launched at the main site University College Hospital (London) (UCH) on the fourth of December 2013 and is currently recruiting at five centres – UCH, Leeds Teaching Hospitals, Ninewells Hospital (Dundee), Northwick Park and St Mark’s Hospitals, Oxford University Hospitals, Queen Alexandra Hospital (Portsmouth). Other sites are currently undergoing site-specific initiations. The trial has recruited 46 patients – 14 in the New diagnosis arm and 32 in the relapse arm.

## Abbreviations

BaF: Barium follow through; CD: Crohn’s disease; CRF: Case report form; CRP: C Reactive protein; CT: Computed tomography; FC: Faecal calprotectin; GCP: Good clinical practice; HAI: Histological activity index; HBI: Harvey bradshaw index; MRE: Magnetic resonance enterography; NHS: National health service; REC: Research ethics committee; T: Tesla; UCH: University College Hospital (London); USS: Ultrasound scan.

## Competing interests

The authors declare that they have no competing interests.

## Authors’ contributions

STa is the principal investigator who conceived, developed and finalised the study. He drafted and finalised the manuscript. SMa is the study statistician who conceived, developed and finalised the study. She drafted and finalised the manuscript. GB is the research fellow attached to the study who contributed to development of sub-studies and drafted and finalised the manuscript. SB contributed to developing the study concept and final study design. He read and approved the final manuscript. AG contributed to developing the study concept and final study design. He read and approved the final manuscript. SH conceived, developed and finalised the study. He read and approved the final manuscript. JH developed and finalised the study. He read and approved the final manuscript. AHa developed and finalised the study. She read and approved the final manuscript. AHi developed and finalised the study. He read and approved the final manuscript. IJ is the patient representative on the study and developed and finalised the study. He read and approved the final manuscript. SMc developed and finalised the study. She read and approved the final manuscript. SMo developed and finalised the study. He read and approved the final manuscript. NM is the head of the clinical trials unit co-ordinating the study and developed and finalised the study. She read and approved the final manuscript. CM developed and finalised the study. He read and approved the final manuscript. SP developed and finalised the study. He read and approved the final manuscript. MJ-R developed and finalised the study. He read and approved the final manuscript. AS developed and finalised the study. He read and approved the final manuscript. STr developed and finalised the study. He read and approved the final manuscript. DT developed and finalised the study. He read and approved the final manuscript. AW developed and finalised the study. He read and approved the final manuscript. PW developed and finalised the study. He read and approved the final manuscript. IZ developed and finalised the study. He read and approved the final manuscript. All authors agree to be accountable for all aspects of the work in ensuring that questions related to the accuracy or integrity of any part of the work are appropriately investigated and resolved. All authors read and approved the final manuscript.

## Pre-publication history

The pre-publication history for this paper can be accessed here:

http://www.biomedcentral.com/1471-230X/14/142/prepub
